# Supplementary material for: Adenovirus protein VII binds the A-box of HMGB1 to repress interferon responses
Source: PLoS Pathog. 2023 Sep 13;19(9):e1011633. doi: 10.1371/journal.ppat.1011633 (PMC10519595; doi:10.1371/journal.ppat.1011633)

Supplemental Table S1

GENOTYPE	PHENOTYPE
pUT18C pKT25	-
pUT18C-GyrB pKT25-GyrA	+++
pUT18-TrcR pKNT25-GapR	-
pUT18C-HMGB1(Abox) pKT25	-
pUT18C-HMGB1 (AB) pKT25	-
pUT18C-HMGB1 (Bbox) pKT25	-
pUT18C-HMGB1 (BC) pKT25	-
pUT18C-HMGB1 pKT25	-
pUT18C pKT25-protein VII	-
pUT18C-HMGB1(Abox) pKT25-protein VII	++++
pUT18C-HMGB1 (AB) pKT25-protein VII	+++/++++
pUT18C-HMGB1 (Bbox) pKT25-protein VII	-
pUT18C-HMGB1 (BC) pKT25-protein VII	-
pUT18C-HMGB1 pKT25-protein VII	++
pUT18C-HMGB1(Abox DNA bending mutant) pKT25	-
pUT18C-HMGB1(AB DNA bending mutant) pKT25	-
pUT18C-HMGB1(DNA bending mutant) pKT25	-
pUT18C-HMGB1(Abox DNA bending mutant) pKT25-protein VII	+++
pUT18C-HMGB1(AB DNA bending mutant) pKT25-protein VII	++
pUT18C-HMGB1 (DNA bending mutant) pKT25-protein VII	++
pUT18C pKT25-VIIΔPTM	-
pUT18C-HMGB1(Abox) pKT25-VIIΔPTM	++
pUT18C-HMGB1 (AB) pKT25-VIIΔPTM	+
pUT18C-HMGB1 (Bbox) pKT25-VIIΔPTM	-
pUT18C-HMGB1 (BC) pKT25-VIIΔPTM	-
pUT18C-HMGB1 pKT25-VIIΔPTM	-
pUT18C pKT25-pVII	-
pUT18C-HMGB1(Abox) pKT25-pVII	+++
pUT18C-HMGB1 (AB) pKT25-pVII	++/+++
pUT18C-HMGB1 (Bbox) pKT25-pVII	-
pUT18C-HMGB1 (BC) pKT25-pVII	-
pUT18C-HMGB1 pKT25-pVII	-
pUT18 pKT25	-
pUT18-HMGB1 (Abox) pKT25	-
pUT18-HMGB1 (BC) pKT25	-
pUT18-HMGB1 pKT25	-
pUT18 pKT25-protein VII	-
pUT18-HMGB1 (Abox) pKT25-protein VII	++
pUT18-HMGB1 (BC) pKT25-protein VII	-
pUT18-HMGB1 pKT25-protein VII	-
pUT18 pKNT25	-

pUT18-HMGB1 (Abox) pKNT25	-
pUT18-HMGB1 (BC) pKNT25	-
pUT18-HMGB1 pKNT25	-
pUT18 pKNT25-protein VII	-
pUT18-HMGB1 (Abox) pKNT25-protein VII	++
pUT18-HMGB1 (BC) pKNT25-protein VII	-
pUT18-HMGB1 pKNT25-protein VII	-
pUT18C pKNT25	-
pUT18C-HMGB1 (Abox) pKNT25	-
pUT18C-HMGB1 (BC) pKNT25	-
pUT18C-HMGB1 pKNT25	-
pUT18C pKNT25-protein VII	-
pUT18C-HMGB1 (Abox) pKNT25-protein VII	-
pUT18C-HMGB1 (BC) pKNT25-protein VII	-
pUT18C-HMGB1 pKNT25-protein VII	-
pUT18C-protein VII pKT25	-
pUT18C-protein VII pKT25-protein VII	-/+
pUT18C-protein VII pKNT25	-
pUT18C-protein VII pKNT25-protein VII	-
pUT18-protein VII pKT25	-
pUT18-protein VII pKT25-protein VII	-
pUT18-protein VII pKNT25	-
pUT18-protein VII pKNT25-protein VII	-
pUT18C-protein VII pKNT25-HMGB1 (Abox)	++
pUT18C-protein VII pKNT25-HMGB1 (BC)	-
pUT18C-protein VII pKNT25-HMGB1	-
pUT18C-protein VII pKT25-HMGB1 (Abox)	+++
pUT18C-protein VII pKT25-HMGB1 (BC)	-
pUT18C-protein VII pKT25-HMGB1	++
pUT18C-HMGB1 (Abox) pKNT25-HMGB1 (Abox)	++
pUT18C-HMGB1 (Abox) pKNT25-HMGB1 (BC)	-
pUT18C-HMGB1 (Abox) pKNT25-HMGB1	-
pUT18C-HMGB1 (Abox) pKT25-HMGB1 (Abox)	-
pUT18C-HMGB1 (Abox) pKT25-HMGB1 (BC)	-
pUT18C-HMGB1 (Abox) pKT25-HMGB1	-
pUT18-HMGB1 (Abox) pKNT25-HMGB1 (Abox)	-
pUT18-HMGB1 (BC) pKNT25-HMGB1 (BC)	-
pUT18-HMGB1 pKNT25-HMGB1	-
pUT18C-HMGB1 (BC) pKNT25-HMGB1 (BC)	-
pUT18C-HMGB1 pKNT25-HMGB1	-
pUT18C-HMGB1 (AB) pKT25-HMGB1 (AB)	-
pUT18C-HMGB1 (Abox) pKT25-HMGB1 (Bbox)	-
pUT18C-HMGB1 (AB) pKT25-HMGB1 (Bbox)	-

pUT18C-HMGB1 (Bbox) pKT25-HMGB1 (Bbox)	-
pUT18C-HMGB1 (Abox DNA bending mutant) pKT25-HMGB1 (Abox DNA bending mutant)	-
pUT18C-HMGB1 (AB DNA bending mutant) pKT25-HMGB1 (AB DNA bending mutant)	-
pUT18C-HMGB1 (DNA bending mutant) pKT25- HMGB1 (DNA bending mutant)	-
pUT18C-VIIΔPTM pKT25-VIIΔPTM	+
pUT18C-pVII pKT25-pVII	-

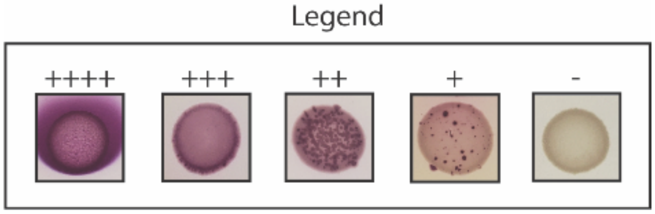

Supplement: S1 Table — Each interaction was tested using four biological replicates. Legend with representative images for each phenotype is provided at the bottom. Positive interactions are indicated in green. (PDF) [file ppat.1011633.s001.pdf]
