# Supplementary figures and images for: Adenovirus protein VII binds the A-box of HMGB1 to repress interferon responses
Source: PLoS Pathog. 2023 Sep 13;19(9):e1011633. doi: 10.1371/journal.ppat.1011633 (PMC10519595; doi:10.1371/journal.ppat.1011633)

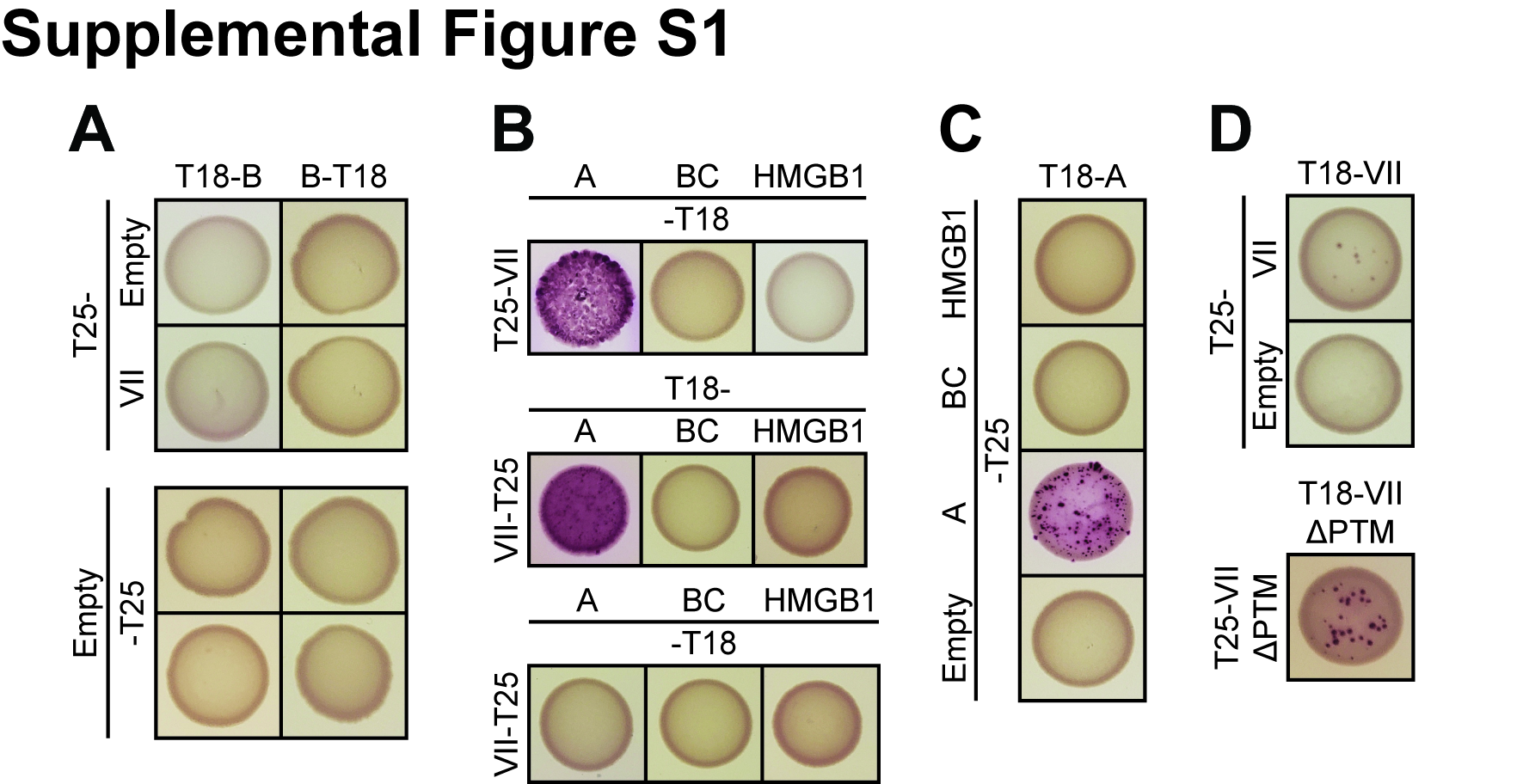

Supplement: S1 Fig — A. B2H assay of HMGB1 B-box in different orientations with protein VII. B. B2H assay between protein VII and indicated HMGB1 variants in three additional orientations. C. B2H assay of HMGB1 A-box with indicated HMGB1 variants showing self-interaction in one instance. D. B2H assay of protein VII N-terminal T25- and N-terminal T18-fusions (top) and an assay of protein VII mutants with alanine substitutions to PTM sites (VII∆PTM) N-terminal T25- and N-terminal T18-fusions (bottom). Both assays show modest self-association. For results of interactions and controls in additional orientations, see S1 Table. (TIF) [file ppat.1011633.s002.tif]

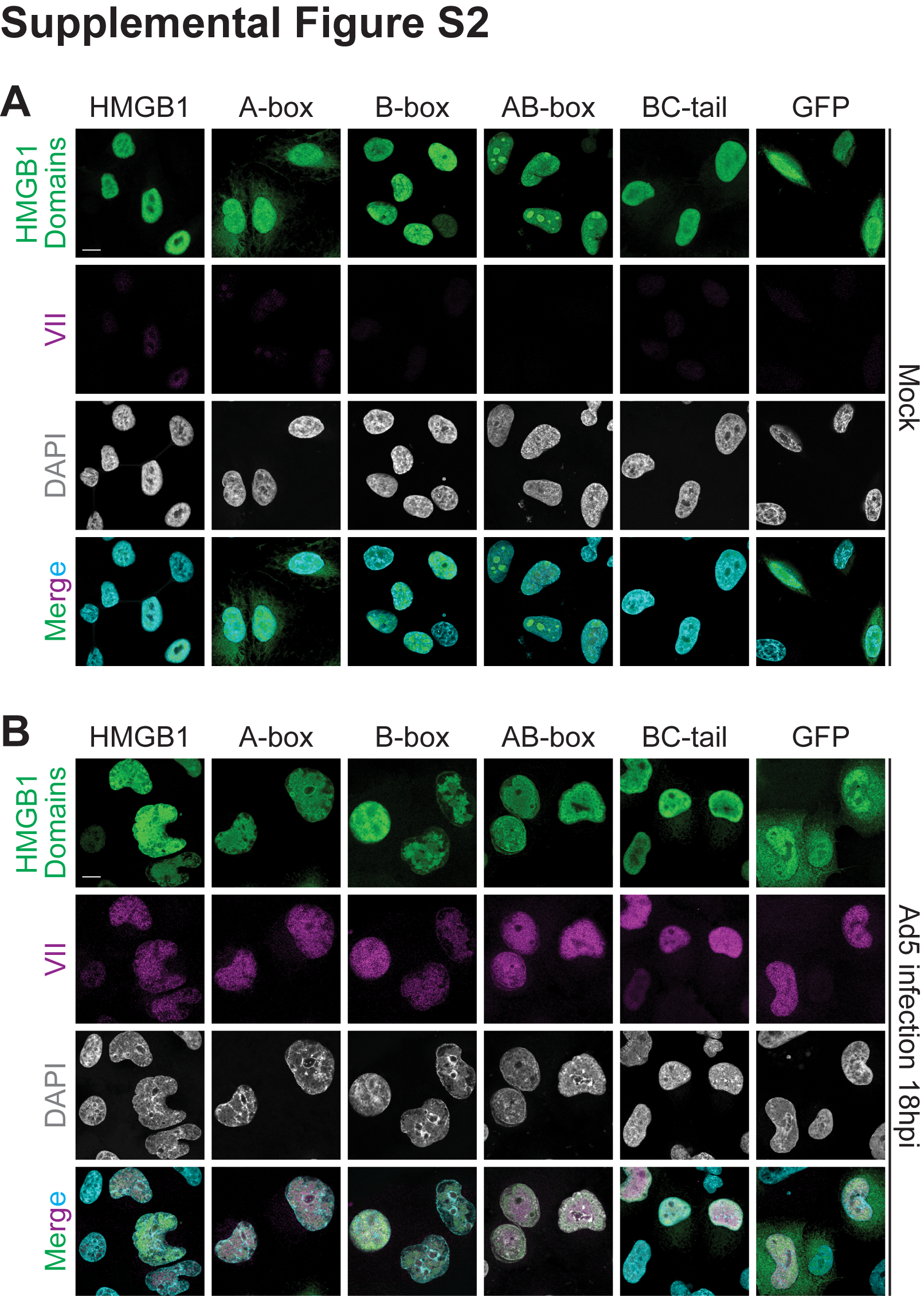

Supplement: S2 Fig — A. Immunofluorescence images showing all channels of mock infected cells from Fig 2C with HMGB1 in green, protein VII in magenta, and DAPI in gray (cyan in merge). Scale bar is 10 μm. B. Immunofluorescence images of Ad5 infected cells showing all channels from Fig 2C with HMGB1 in green, protein VII in magenta, and DAPI in gray (cyan in merge). Scale bar is 10 μm. (TIF) [file ppat.1011633.s003.tif]

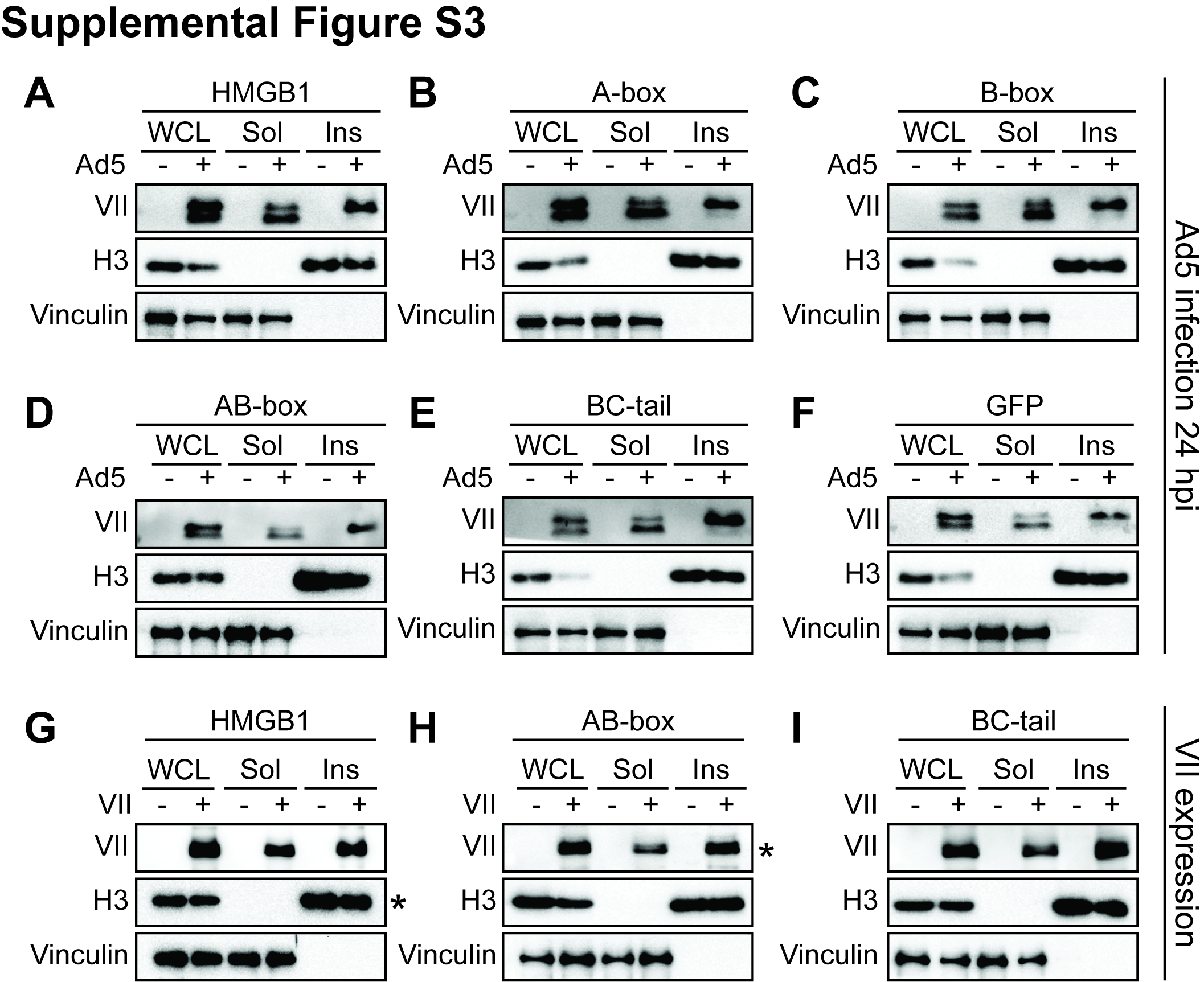

Supplement: S3 Fig — Western blots showing all controls that correspond with Fig 3C for protein VII, H3, GFP, and vinculin: HMGB1 (A), A-box (B), B-box (C), AB-box (D), BC-tail (E), and GFP (F). Western blots showing all controls that correspond with Fig 3E for protein VII, H3, GFP, and vinculin; HMGB1 (G), AB-box (H), and BC-tail (I). (TIF) [file ppat.1011633.s004.tif]

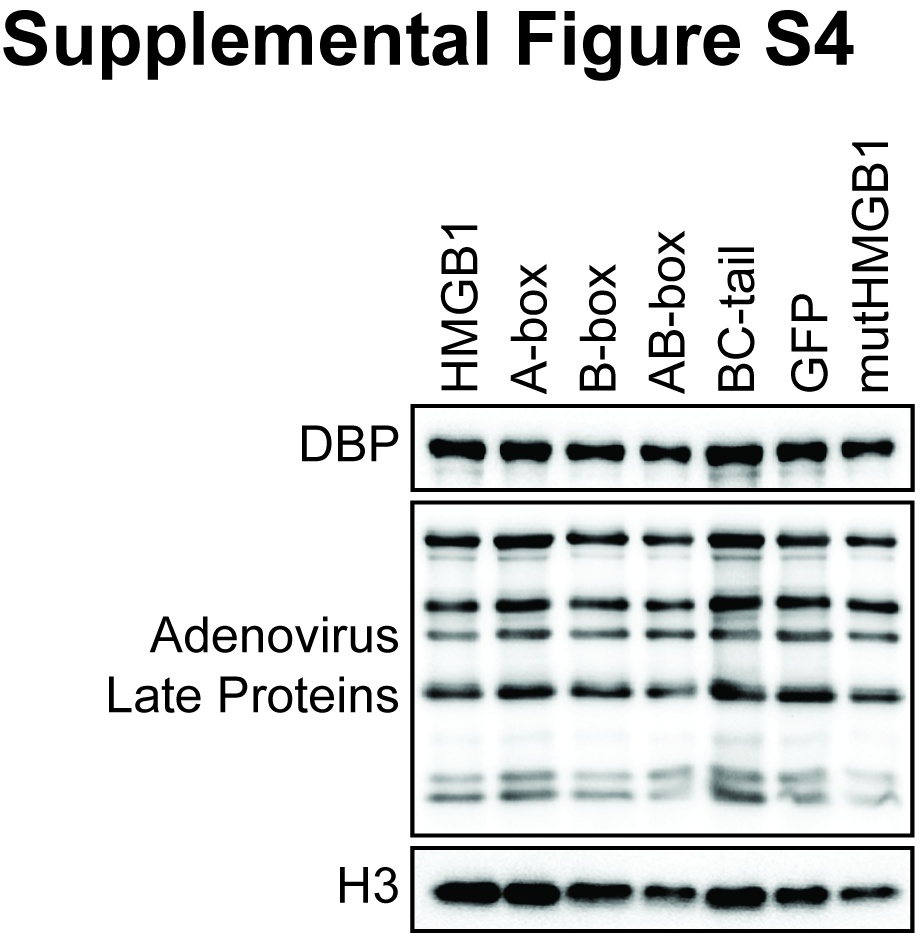

Supplement: S4 Fig — (TIF) [file ppat.1011633.s005.tif]
