# Supplementary material for: Adenovirus protein VII binds the A-box of HMGB1 to repress interferon responses
Source: PLoS Pathog. 2023 Sep 13;19(9):e1011633. doi: 10.1371/journal.ppat.1011633 (PMC10519595; doi:10.1371/journal.ppat.1011633)
Supplement: Attachment — Submitted filename: PLoS Pathogens Reviewer Response - 2023-8-17.pdf [file ppat.1011633.s007.pdf]

PLoS Pathogens Review August 17, 2023

The reviewers were please with the manuscript revisions. There remains a few lingering issues, most notably with Figure 7B. It would appear that alterations to the text should be all that is required to mitigate these concerns. The authors are encouraged to address all of the comments form the reviewers. A final submission of the manuscript would be editorially evaluated rapidly.

Reviewer Comments (if any, and for reference):

Reviewer's Responses to Questions

Part I - Summary
Please use this section to discuss strengths/weaknesses of study, novelty/significance, general execution and scholarship.

Reviewer #1: Arnold et al have resubmitted a repaired version of their continuing studies of the interaction of HAdV pVII with HMGB1. The list of minor corrections I provided have been addressed. I am happy with the improved quantification and the use of a better control for the pVII overexpressing virus. I don't think any additional mechanistic details have been teased out, but I was already happy with the extent of the data provided in the initial manuscript.

Reviewer #2: The authors previously demonstrated that adenovirus core protein VII, a histone-like viral protein, binds the high mobility group protein HMGB1 and sequesters this protein to chromatin. This process prevents HMGB1 from being released from cells and disrupts its ability to function as an alarmin to promote inflammatory signaling. In this submission, the authors characterize this process further. Using a bacterial one-hybrid approach followed by immunofluorescence microscopy in A549 cells, they demonstrate that protein VII binds to the A domain of HMGB1. Protein VII sequestration of HMGB1 results in ~75% of the HMGB1 pool fractionating in an insoluble fraction; the HMGB1 A box is necessary and sufficient for this to occur. HMGB1 DNA binding is not required. The authors previously showed that protein VII is post-translationally modified and this contributes to chromatin association. Here they show that VII PTMs are not required for HMGB1 binding but are required to confer insolubility. Finally, the authors show that protein VII expression does not inhibit an interferon-beta response, but does repress the induction of interferon-beta expression by poly(dAdT).

The manuscript is clearly written and overall the data are convincing. The manuscript does not address underlying mechanisms of activity (why VII PTMs are not required to relocalize HMGB1 but are required to induce HMGB1 insolubility, how VII regulates the induction of interferon-beta expression by polydAdT), thus the impact of the study is limited.

Reviewer #3: The strength of this report is the novel methods used to study the biochemical properties of a key adenovirus chromatin protein and cellular regulator. Previous weaknesses

have been rectified and appropriate statistical and quantitative measures are provided that justify the conclusions.

Part II – Major Issues: Key Experiments Required for Acceptance
Please use this section to detail the key new experiments or modifications of existing experiments that should be absolutely required to validate study conclusions.

Generally, there should be no more than 3 such required experiments or major modifications for a "Major Revision" recommendation. If more than 3 experiments are necessary to validate the study conclusions, then you are encouraged to recommend "Reject".

Reviewer #1: none

Reviewer #2: (No Response)

Reviewer #3: None required.

Part III – Minor Issues: Editorial and Data Presentation Modifications
Please use this section for editorial suggestions as well as relatively minor modifications of existing data that would enhance clarity.

Reviewer #1: 1) Line 188: Generally the word serotype is not used with regards to adenovirus anymore. This should just be "type", or more commonly HAdV-C5.

We thank the reviewer for these suggestions. We have made this change to the text.

2) Line 341: Not sure about the jump to cell cycle here. Seems like a big speculative leap without any basis.

We agree with the reviewer and have removed the claim from the manuscript.

3) Line 382: The sentence starting with "Strikingly" is a bit confusing placed where it is. The strikingly would be better moved to describe the complete abrogation of basal INFbeta expression by pVII in the deltaHMGB1 cells not treated with poly(dA:dT). I found that striking.

We agree with the reviewer and have made this change to the text.

4) Some of the journal titles in the refs seem a little off the normal abbreviations. What's with the dagger symbol in ref 44 line 833?

We agree and have removed the non-standard symbol and changed the journal titles to their standard abbreviations.

Reviewer #2: Fig. 7 is improved with the required Ad-GFP control virus, but there are two aspects in 7B that require clarification. First, the authors state in lines 384-387: "In the absence of HMGB1 (A549ΔHMGB1 cells), we found a robust increase in IFNβ expression upon stimulation with poly(dA:dT) that reached significantly higher levels than those of the GFP expressing A549 cells." The figure shows that the statistical significance is higher (P = <.05 vs p = <.005) but the magnitude of the difference between the two cell lines does not appear to be that great. If the authors want to make this claim, they need to state the numerical differences between the Ad-GFP +/- poly(dA:dT) in the A549 vs. A549-dlHMGB1 cells.

We agree with the reviewer that Fig 7B requires further explanation. We addressed the first issue by reviewing the claim from the text of the manuscript as follows: while the difference between poly(dA:dT) A549 cells with rAd-GFP and A549ΔHMGB1 cells with rAd-GFP is statistically significant, the magnitude of the difference is small and likely not biologically significant.

Second, the A549-dlHMGB1 cells + Ad-VII-GFP, No Treatment, sample (7th bar) shows a complete loss of IFNbeta expression compared to the Ad-GFP control, and compared to the two No Treatment samples in A549 cells (left side). There is no explanation given for this result. What is the level of IFNbeta expression in uninfected A549 cells? Do the levels seen in Ad-GFP and Ad-VII-GFP infected cells, No Treatment, represent an induction of IFNbeta expression compared to control, uninfected cells? Also, given the greater than 5 log difference in Ad-VII-GFP infected A549dlHMGB1 with No Treatment vs. poly(dA:dT), how can the significance only be <.05? Something is amiss here and it certainly requires an explanation and/or modification.

We appreciate the reviewers nuanced reading of this figure and pointing out where we could be clearer in our explanation. The issue raised regarding the low p value is due to the large variance of IFN expression upon induction and how the data was normalized. Since the IFNbeta droplets are normalized by back-calculating the dilution factor of the cDNA (100-fold) and then dividing by every 1 million positive 18S droplets, a very low number of positive IFNbeta droplets (e.g. 1-5) could result in a slightly skewed result. In the case of poly(dA:dT) A549ΔHMGB1 cells with rAd VII-GFP, all three values were 0, thus why the value is extremely low compared to other values, resulting in a low p-value despite the biological significance. To address this issue, we have included an explanation in the text explaining the normalization and, importantly, we have added a dotted line at 10^2 to delineate the baseline levels of IFNbeta expression.

Reviewer #3: None identified.
