# Supplementary material for: Adenovirus protein VII binds the A-box of HMGB1 to repress interferon responses
Source: PLoS Pathog. 2023 Sep 13;19(9):e1011633. doi: 10.1371/journal.ppat.1011633 (PMC10519595; doi:10.1371/journal.ppat.1011633)
Supplement: Attachment — Submitted filename: Reviewer Response_PLoS Pathogens Reviews_final.docx [file ppat.1011633.s006.docx]

**PLoS Pathogens Reviews May 11, 2023**

CC: "Edward Arnold" [earnold4@uw.edu](mailto:earnold4@uw.edu), "Robin Kaai" [rkaai@uw.edu](mailto:rkaai@uw.edu), "Katie Leung" [kleung2@uw.edu](mailto:kleung2@uw.edu), "Mia Brinkley" [mbrinkle@fredhutch.org](mailto:mbrinkle@fredhutch.org), "Laurel Kelnhofer-Millevolte" [lkelnhof@fredhutch.org](mailto:lkelnhof@fredhutch.org), "Monica Guo" [msguo@uw.edu](mailto:msguo@uw.edu)

Dear Dr. Avgousti,

Thank you very much for submitting your manuscript "Adenovirus protein VII binds the A-box of HMGB1 to repress interferon responses." for consideration at PLOS Pathogens. As with all papers reviewed by the journal, your manuscript was reviewed by members of the editorial board and by several independent reviewers. In light of the reviews (below this email), we would like to invite the resubmission of a significantly-revised version that takes into account the reviewers' comments.

The reviewers are intrigued by your study but find areas of it deficient in quantitation, statistical analyses, and the implementation of controls.

We cannot make any decision about publication until we have seen the revised manuscript and your response to the reviewers' comments. Your revised manuscript is also likely to be sent to reviewers for further evaluation.

When you are ready to resubmit, please upload the following:

[1] A letter containing a detailed list of your responses to the review comments and a description of the changes you have made in the manuscript. Please note while forming your response, if your article is accepted, you may have the opportunity to make the peer review history publicly available. The record will include editor decision letters (with reviews) and your responses to reviewer comments. If eligible, we will contact you to opt in or out.

[2] Two versions of the revised manuscript: one with either highlights or tracked changes denoting where the text has been changed; the other a clean version (uploaded as the manuscript file).

Important additional instructions are given below your reviewer comments.

Please prepare and submit your revised manuscript within 60 days. If you anticipate any delay, please let us know the expected resubmission date by replying to this email. Please note that revised manuscripts received after the 60-day due date may require evaluation and peer review similar to newly submitted manuscripts.

Thank you again for your submission. We hope that our editorial process has been constructive so far, and we welcome your feedback at any time. Please don't hesitate to contact us if you have any questions or comments.

Sincerely,

Robert F. Kalejta
Academic Editor
PLOS Pathogens

Blossom Damania
Section Editor
PLOS Pathogens

Kasturi Haldar
Editor-in-Chief
PLOS Pathogens
[orcid.org/0000-0001-5065-158X](http://orcid.org/0000-0001-5065-158X)

Michael Malim
Editor-in-Chief
PLOS Pathogens
[orcid.org/0000-0002-7699-2064](http://orcid.org/0000-0002-7699-2064)

***********************

The reviewers are intrigued by your study but find areas of it deficient in quantitation, statistical analyses, and the implementation of controls.


Reviewer's Responses to Questions

**Part I - Summary**
Please use this section to discuss strengths/weaknesses of study, novelty/significance, general execution and scholarship.

Reviewer #1: Arnold et al report further studies characterizing the interaction of HAdV pVII with HMGB1. Bacterial 2-hybrid tests are done to demonstrate direct interactions and map interaction domains. A HMGB1 KO line is used for some interesting reconstitution studies looking at pVII effects on HMGB1 localization, again mapping domains and looking at the impact of mutations affecting known post-translational modification. This data includes a solubility assay that I thought was quite nice as surrogate for tight chromatin association. Ultimately, a convincing effect on repression of IFNb expression by pVII is demonstrated, which is an exciting new finding.

Reviewer #2: The authors previously demonstrated that adenovirus core protein VII, a histone-like viral protein, binds the high mobility group protein HMGB1 and sequesters this protein to chromatin. This process prevents HMGB1 from being released from cells and disrupts its ability to function as an alarmin to promote inflammatory signaling. In this submission, the authors characterize this process further. Using a bacterial one-hybrid approach followed by immunoflouresence microscopy in A549 cells, they demonstrate that protein VII binds to the A domain of HMGB1. Protein VII sequestration of HMGB1 results in ~75% of the HMGB1 pool fractionating in an insoluble fraction; the HMGB1 A box is necessary and sufficient for this to occur. HMGB1 DNA binding is not required. The authors previously showed that protein VII is post-translationally modified and this contributes to chromatin association. Here they show that VII PTMs are not required for HMGB1 binding but are required to confer insolubility. Finally, the authors show that protein VII expression does not inhibit an interferon-beta response, but does repress the induction of interferon-beta expression by poly(dAdT).

The manuscript is clearly written and overall the data are convincing. The manuscript does not address underlying mechanisms of activity (why VII PTMs are not required to relocalize HMGB1 but are required to induce HMGB1 insolubility, how VII regulates the induction of interferon-beta expression by polydAdT), thus the impact of the study is limited.

Reviewer #3: Previous research by Dr. Avgousti and associates has established a remarkable relationship between the major adenovirus "chromatin" protein, protein VII, and the cellular high-mobility group box 1 (HMGB1) "chromatin" protein. This relationship is of interest and is significant because protein VII has been shown to dampen the cellular anti-viral response. This has been shown to be due, at least in part, to the action of protein VII on HMGB1. This curious chromatin-associate protein is released by damaged cells where it acts as a chemokine or cytokine to signal danger. This makes understanding the nature of both of these part-time chromatin proteins important.

Using a rather comprehensive collection of constructs to study protein-protein interactions in bacteria, it was convincingly shown that one of the two DNA-binding domains in HMGB1 (the A-box) directly interacts with protein VII and that this can occur in the absence of posttranslational modifications known to affect protein VII function and in the absence of the DNA-binding property of HMGB1. Another intriguing, but inadequately supported observation is that this interaction likely results in the retention of HMGB1 in the nucleus of the infected cell. Although the interaction between HMGB1 and protein VII does not require posttranslational modifications to protein VII, these modifications may be important for the capacity of protein VII to render HMGB1 less freely soluble.

The strength of this report is that the findings in bacteria are clear and convinced and--if substantiated--the findings observed in the eukaryotic cell would be important. A significant limitation to this report is the failure to adequately quantify and validate the localization findings described in several figures.

The presentation, writing and clarity of the report is solid.

**Part II – Major Issues: Key Experiments Required for Acceptance**
Please use this section to detail the key new experiments or modifications of existing experiments that should be absolutelyrequired to validate study conclusions.

Generally, there should be no more than 3 such required experiments or major modifications for a "Major Revision" recommendation. If more than 3 experiments are necessary to validate the study conclusions, then you are encouraged to recommend "Reject".

Reviewer #1: none

Reviewer #2: The results in Figs. 6B/C are difficult to interpret. HMGB1 expression alone reduces the induction of interferon-beta expression ~5-fold (estimated by this reviewer from 6B) and protein VII alone reduces interferon-beta expression with control GFP 10-fold (quantified in 6C). The net change when both HMGB1 and VII are expressed is an additional 4-fold decrease in interferon-beta expression (6C). This is a statistically significant but rather modest effect. What is missing from this experiment is a control adenovirus vector to control for Ad-VII infection, ie. that the adenovirus vector infection does not contribute to the observed effect. Also, if HMBG1 and VII coordinate to inhibit the induction of interferon-beta expression by poly(dAdT), then one would anticipate that this should be found with other inducers of interferon-beta expression such as poly(IC) and RNA virus infection.

*We agree with the reviewer that a control recombinant adenovirus is needed and that our initial statistics could have been better explained. Because qPCR is a somewhat variable technique, to repeat this experiment more rigorously, we turned to droplet digital PCR that is a much more reliable and reproducible method. We repeated the experiment as originally presented but with the additional treatment of a control recombinant adenovirus vector (rAd GFP). Additionally, to avoid further variability introduced by our transgenic cell lines, we used A549 and A549∆HMGB1 cells only that either have endogenous levels of HMGB1, or none. The results presented in the new Figure 7 show that protein VII inhibits interferon-beta expression in an HMGB1-dependent manner. Without HMGB1, protein VII can still depress interferon-beta expression, but not to the same degree. Furthermore, we see no inhibition of interferon-beta induction in our poly(dA:dT) treated cells transduced with the control adenovirus vector (rAd GFP), indicating that the recombinant adenovirus vector is not contributing to the inhibition of interferon-beta. Therefore, we conclude that protein VII has a specific effect on interferon-beta that is dependent on HMGB1. These data are presented in revised Figure 7B.*

*While we agree with the reviewer that other inducers of interferon-beta should cause the same effect in theory, because an RNA virus would be less biologically relevant to our system and due to time constraints, we chose to focus on the necessary control for our system. Future work will investigate the robustness of protein VII’s effect on IFN with other inducers.*

Reviewer #3: HMGB1, which is normally released from cells treated with the detergent NP-40, is retained in an insoluble form by protein VII through the A-box. It seems likely that this finding is significant but this was not appropriately demonstrated. First, the results in Fig. 2C are not convincing. If the difference being highlighted is differential localization within the nucleus, this should be systematically quantified and reported. The small sampling of examples in Fig. 2C display a rather broad diversity of localization patterns with insignificant numbers of examples to warrant any definitive conclusions. Similarly, the four and one-half cells shown in Fig. 5C inadequately support the conclusions. There are well-established means of selectively solubilizing cells while preserving the morphological integrity of the cell. Application of these methods together with more rigorous quantitative image analysis would likely address this shortcoming and provide valuable insight.

*We agree with the reviewer that a deeper examination by immunofluorescence is warranted support our findings. To address this, we have systematically quantified the patterns observed in Figure 2. We scored blindly and binned the patterns into three distinct groups as described in the new Figure 2. These results, presented in the revised Figure 2D, clearly show that the patterns of HMGB1 are mostly affected by adenovirus infection when the A-box is present and to a slight degree with the B box. To address the reviewer’s concerns specifically with respect to solubility, we have taken the reviewers suggestion and repeated these experiments with the addition of pre-extraction prior to fixation. This method has been well established to release soluble proteins and retain those that are more insoluble, allowing a clearer of view changes in solubility (e.g. Reyes et al MCP 2017). Our new results are striking and unambiguously demonstrate that A-box containing constructs of HMGB1 are retained within the nucleus during adenovirus infection and that those lacking A-box are released during pre-extraction, resulting in no signal. These data are presented in the revised Figure 3.*

A second concern regards the use of the E1-deleted recombinant adenovirus to express protein VII. Replication-defective adenovirus vectors express more than just the transgene. The E4 gene products are expressed at non-trivial levels and E4orf1, E4orf3 and E4orf6 have been reported to have biological effects at levels nearly impossible to detect (see for example Ramalingam, 1999, [https://doi.org/10.1182/blood.V93.9.2936](https://urldefense.com/v3/__https:/doi.org/10.1182/blood.V93.9.2936__;!!GuAItXPztq0!kAE1Oz7gFyQaC_o4p-Qq1x5XOmLY857YdSNlcgYVV7mXPvZlFNm3YtbLy4IcRF51C3rTHgxiLQayG3bjcqE$)). An appropriate control for the VII-expressing recombinant virus would be one that expresses an irrelevant gene. Was this done? If so, the results need to be reported. If not, this control needs to be added.

*We agree with the reviewer that a control recombinant adenovirus is needed, as noted by reviewer 2 above. We have now repeated the experiment as originally presented but with the additional treatment of a control recombinant adenovirus vector (rAd GFP). As stated above, to avoid further variability introduced by our transgenic cell lines, we used A549 and A549∆HMGB1 cells only that either have endogenous levels of HMGB1, or none. The results presented in the new Figure 7 show that protein VII inhibits interferon-beta expression in an HMGB1-dependent manner. Without HMGB1, protein VII can still depress interferon-beta expression, but not to the same degree. Furthermore, we see no inhibition of interferon-beta induction in our poly(dA:dT) treated cells transduced with the control adenovirus vector (rAd GFP), indicating that the recombinant adenovirus vector is not contributing to the inhibition of interferon-beta. Therefore, we conclude that protein VII has a specific effect on interferon-beta that is dependent on HMGB1. These data are presented in revised Figure 7B.*

The third issue is that the statistical tests applied here were not adequately described. What specific tests in Prism were used? What was the correction for the multiple comparisons reported in several figures? If parametric tests were used for the statistical analysis, were the residuals appropriate for these tests? If so, report this.

*We agree with the reviewer that the description of statistics was inadequate in our original submission and have now thoroughly described this in the figure legends and methods section. Specifically, we have used a Welch’s t-test when comparing the densitometry values for western blots in revised Figures 4D, 4F, and 5F. We used a Student’s t-test in revised Figure 7A and 7B. These are now included in the figure legends, as well as stating p and n values for each experiment.*

**Part III – Minor Issues: Editorial and Data Presentation Modifications**
Please use this section for editorial suggestions as well as relatively minor modifications of existing data that would enhance clarity.

Reviewer #1: Minor comments relate to the data:
For figure 2 and any others with confocal microscopy, calculating overlap in signals for HMGB1 and pVII with a Pearson's correlation for statistical significance would be appropriate. Similarly, this can be done for fraction of HMGB1 at various subcellular locations, like the nucleolus.
Putting standard deviation significance indications on the bar graphs would be a good idea.

*We thank the reviewer for this suggestion and agree that further analysis was needed. Unfortunately, a Pearson’s correlation would show overlap for all conditions as these proteins are highly abundant and globally nuclear, despite varying patterns in different conditions. However, we agree with Reviewers 1 and 2 that quantification combined with pre-extraction of our samples prior to microscopy analysis would address this concern. Therefore, we have now carried out quantification of the patterns observed in Figure 2 and repeated these experiments with the addition of pre-extraction prior to fixation. Please see the response to Reviewer 2 above and revised Figure 2D and revised Figure 3. We further agree that our statistical descriptions were lacking and have now added more description to our figure legends and the methods section.*

Minor textual suggestions:
I believe that all the HAdV reagents here are from HAdV-C5, but it wasn't specified anywhere that I noticed other than for describing the virus infection experiments.
Line 296 - rather than say "present in human cells", it might be better to say mammalian or eukaryotic cells.
Lines 350-351 - the HMGB1 independent effects of pVII on IFNb expression are very interesting. Is it possible that pVII can bind another HMGB protein (like HMGB2) in the absence of HMGB1 that has overlapping functions?
Line 460 - depletes sounds a little awkward here. Would limits or restricts be a better choice?
-The source of the A549 HMGB1 KO cells is not explicitly described in the methods.
-Some of the title of the methods sections have inconsistent internal capitalization compared to the others.

*We agree with the reviewer on all points and have amended the text accordingly.*

Reviewer #2: Line 55 - the literature (ref 9) shows that protein VII is released from the adenovirus gene during the early phase of infection.
Line 140 - more accurately state that protein VII is cleaved during the process of virion maturation.

*We agree with the reviewer and have amended the text accordingly.*

The GFP images are shown in black and white - I think they would be easier to visualize in color and the merge images show GFP in color.

*We agree with the reviewer and have changed to the corresponding pseudocolors in all figures. Please see new Figures 2, 5, and 6.*

Fig. 4E - how is a value >1 obtained (ie. more HMBG1 is insoluble than present in the control lysate)?

*We agree that this result was not clear as originally described. There is some variability from sample to sample and, importantly, this slight increase is not statistically significant. To clarify for the reviewer, we normalize to the amount of HMGB1 visualized by WB from the whole cell lysate of each sample. In the case of the mutHMGB1, the concentration per volume loaded of mutHMGB1 was greater than WT HMGB1 in this experiment. This is normal variability of this assay and is not biologically meaningful.*

Fig. 5B - I question if the results with the VII-PTM mutant in the bacterial one-hybrid assay should even be included in the manuscript. These modifications will not occur in bacteria with wild-type VII so what is the point?

*While we agree that the modifications will not occur in bacteria, it is not yet known whether they also provide a structural function for protein VII. We observed in yeast that this mutant was unable to disrupt the cell cycle will the WT protein was (Lynch et al Curr Bio 2021), even though it is likely that these residues were also not modified in yeast. We have included this in the discussion and feel that it is an important point for future structural studies to reference that these sites may play a more important role in 3 dimensions. Please see new lines 420-424.*

Lines 321-322 - the title of this section is not accurate. Fig. 6A shows that protein VII does not inhibit the type-1 interferon response since no effect was observed on the induction of ISGs following interferon-beta addition. Rather, the data indicate that protein VII and HMGB1 inhibit interferon signaling.

*We agree with the reviewer and have changed the title to ‘protein VII inhibits interferon signaling by decreasing IFNβ1 expression’.*

Reviewer #3: The phrase "human cell biological systems" is rather devoid of meaning in the context of the abstract. A more precise term would be better.

*We agree with the reviewer and have changed the phrase to human cell culture to be more accurate.*

While understandable, the term "preVII" has not been the term historically used for precursor adenovirus proteins (although it should have been.) It is helpful in the figures but rather than introduce yet another element of nomenclature, perhaps it should be kept as "pVII" in the text and a note made in the figure legends of this change.

*We agree with the reviewer and have changed the text accordingly.*

Formally speaking, one does not "mutate" hydrophobic residues. It would more correct to simply say that these residues were changed (by a mutation in the gene.)

*We agree with the reviewer and have changed the text accordingly.*

Figure legends generally fail to provide sufficient information to understand the figure. The nature of the error bars is not explained in the legends.

*We agree with the reviewer and have now included more information in each figure legend. More specifically, all error bars represent the standard deviation of the data presented.*

PLOS authors have the option to publish the peer review history of their article ([what does this mean?](https://urldefense.com/v3/__https:/journals.plos.org/plospathogens/s/editorial-and-peer-review-process*loc-peer-review-history__;Iw!!GuAItXPztq0!kAE1Oz7gFyQaC_o4p-Qq1x5XOmLY857YdSNlcgYVV7mXPvZlFNm3YtbLy4IcRF51C3rTHgxiLQay7hGClZc$)). If published, this will include your full peer review and any attached files.

If you choose “no”, your identity will remain anonymous but your review may still be made public.

**Do you want your identity to be public for this peer review?** For information about this choice, including consent withdrawal, please see our [Privacy Policy](https://urldefense.com/v3/__https:/www.plos.org/privacy-policy__;!!GuAItXPztq0!kAE1Oz7gFyQaC_o4p-Qq1x5XOmLY857YdSNlcgYVV7mXPvZlFNm3YtbLy4IcRF51C3rTHgxiLQayE_JpzvE$).

Reviewer #1: No

Reviewer #2: No

Reviewer #3: No

Figure Files:

While revising your submission, please upload your figure files to the Preflight Analysis and Conversion Engine (PACE) digital diagnostic tool, [https://pacev2.apexcovantage.com](https://urldefense.com/v3/__https:/pacev2.apexcovantage.com/__;!!GuAItXPztq0!kAE1Oz7gFyQaC_o4p-Qq1x5XOmLY857YdSNlcgYVV7mXPvZlFNm3YtbLy4IcRF51C3rTHgxiLQay3DI3Rjk$). PACE helps ensure that figures meet PLOS requirements. To use PACE, you must first register as a user. Then, login and navigate to the UPLOAD tab, where you will find detailed instructions on how to use the tool. If you encounter any issues or have any questions when using PACE, please email us at [figures@plos.org](mailto:figures@plos.org).

Data Requirements:

Please note that, as a condition of publication, PLOS' data policy requires that you make available all data used to draw the conclusions outlined in your manuscript. Data must be deposited in an appropriate repository, included within the body of the manuscript, or uploaded as supporting information. This includes all numerical values that were used to generate graphs, histograms etc.. For an example see here on PLOS Boology:[http://www.plosbiology.org/article/info%3Adoi%2F10.1371%2Fjournal.pbio.1001908#s5](https://urldefense.com/v3/__http:/www.plosbiology.org/article/info*3Adoi*2F10.1371*2Fjournal.pbio.1001908*s5__;JSUlIw!!GuAItXPztq0!kAE1Oz7gFyQaC_o4p-Qq1x5XOmLY857YdSNlcgYVV7mXPvZlFNm3YtbLy4IcRF51C3rTHgxiLQayj0AbXjY$).

Reproducibility:

To enhance the reproducibility of your results, we recommend that you deposit your laboratory protocols in [protocols.io](http://protocols.io), where a protocol can be assigned its own identifier (DOI) such that it can be cited independently in the future. Additionally, PLOS ONE offers an option to publish peer-reviewed clinical study protocols. Read more information on sharing protocols at[https://plos.org/protocols?utm_medium=editorial-email&utm_source=authorletters&utm_campaign=protocols](https://urldefense.com/v3/__https:/plos.org/protocols?utm_medium=editorial-email&utm_source=authorletters&utm_campaign=protocols__;!!GuAItXPztq0!kAE1Oz7gFyQaC_o4p-Qq1x5XOmLY857YdSNlcgYVV7mXPvZlFNm3YtbLy4IcRF51C3rTHgxiLQayp3rREKs$)
